# COVID-19, Oxidative Stress and Male Reproduction: Possible Role of Antioxidants

**DOI:** 10.3390/antiox11030548

**Published:** 2022-03-14

**Authors:** Pallav Sengupta, Sulagna Dutta, Shubhadeep Roychoudhury, Urban John Arnold D’Souza, Kadirvel Govindasamy, Adriana Kolesarova

**Affiliations:** 1 Physiology Unit, Faculty of Medicine, Bioscience and Nursing, MAHSA University, Jenjarom 42610, Selangor, Malaysia; pallav@mahsa.edu.my; 2 School of Medical Sciences, Bharath Institute of Higher Education and Research (BIHER), Chennai 600126, India; duttasulagna@mahsa.edu.my; 3 Department of Oral Biology and Biomedical Sciences, Faculty of Dentistry, MAHSA University, Jenjarom 42610, Selangor, Malaysia; 4 Department of Life Science and Bioinformatics, Assam University, Silchar 788011, India; 5 Father Muller Medical College, Mangalore 575025, India; dsouzaurbanjohn@gmail.com; 6 Father Muller College of Allied Health Sciences, Kankanady, Mangalore 575002, India; 7 Animal Production Division, ICAR Research Complex for NEH Region, Indian Council of Agricultural Research, Umiam 793103, India; govindasamy.kadirvel@icar.gov.in; 8 Faculty of Biotechnology and Food Sciences, Slovak University of Agriculture in Nitra, 94976 Nitra, Slovakia; adriana.kolesarova@uniag.sk

**Keywords:** antioxidants, COVID-19, inflammation, male infertility, oxidative stress

## Abstract

Coronavirus disease 2019 (COVID-19) involves a complex pathogenesis and with the evolving novel variants of the severe acute respiratory syndrome coronavirus 2 (SARS-CoV-2), the long-term impacts of the unceasing COVID-19 pandemic are mostly uncertain. Evidence indicates deleterious impact of this disease upon male reproductive health. It is concerning that COVID-19 may contribute to the already global declining trend of male fertility. The adverse impacts of COVID-19 on male reproduction may primarily be attributed to the induction of systemic inflammatory responses and oxidative stress (OS), which operate as a vicious loop. Bringing the systemic inflammation to a halt is critical for ‘putting out’ the ‘cytokine storm’ induced by excessive reactive oxygen species (ROS) generation. The possibility of OS playing a prime role in COVID-19-mediated male reproductive dysfunctions has led to the advocacy of antioxidant therapy. An array of antioxidant defense medications has shown to be effective in experimental and clinical studies of COVID-19. The present review thus discusses the possibilities as to whether antioxidant drugs would contribute to combating the SARS-CoV-2 infection-induced male reproductive disruptions, thereby aiming at kindling research ideas that are needed for identification and treatment of COVID-19-mediated male reproductive impairments.

## 1. Introduction

The severe acute respiratory syndrome coronavirus-2 (SARS-CoV-2), first identified in December 2019 in Wuhan city of China [1], causes the coronavirus disease-2019 (COVID-19). Attributable to its inordinate transmissibility, World Health Organization (WHO) has labelled it as a global ‘pandemic’ on 11 March 2020 [2]. Various hypotheses have already been put forth to explain its pathogenesis. It has been observed that men are more vulnerable to COVID-19 compared to women [3]. Thus, it is essential to focus on the possible mechanisms of COVID-19-mediated impairment of men’s health as well as fertility. There is hardly any more confusion regarding the fact that the virus may enter the testes [4,5,6], presumably due to the infection-mediated inflammatory response disrupting the blood-testes barrier. Furthermore, testicular immunological privilege and the local presence of regulatory T cells may aid viral persistence in this tissue [7]. Li et al. recently reported SARS-CoV-2 presence in semen samples in six individuals, two of whom were recovering from COVID-19. This discovery reopened debates about male genital tract infection, viral shedding in sperm, and prospects of reproductive therapies for COVID-19 patients [5]. The testes of deceased COVID-19 patients showed testicular congestion, interstitial oedema, exudation of red blood cells, and T-lymphocyte (CD3+) and macrophage infiltration. Both the testis and epididymides showed elevated inflammatory responses. The same study found elevated ACE2 expression in the Leydig cells of males who died with COVID-19 [8]. The increase in apoptotic cells inside seminiferous tubules of deceased COVID-19 patients suggests SARS-CoV-2-mediated spermatogenic disruptions. Moreover, the influence of the viral infection on semen quality and endocrine dys-homeostasis is also known. According to Ma et al. [9], COVID-19 patients had higher circulating levels of LH and lower testosterone: LH ratio, indicating impaired Leydig cell activity. Following infection, Koç and Keserolu [10] found a decrease in circulating testosterone levels, which Cinislioglu et al. [11] linked to the severity of the disease. The findings for semen quality are more varied. A substantial influence of COVID-19 infection on semen quality has been reported, including semen volume, total sperm motility, progressive motility, and sperm morphology [10,12,13].

It is noteworthy that the general mode of actions of any respiratory viral infections (that include inflammatory responses, cytokine production, host cell apoptosis, and subsequently a chain of systemic pathophysiological processes) may be attributed to compromised antioxidant actions and the induction of oxidative stress (OS) [14]. Antioxidant treatments for COVID-19 show promising results; thus, it will be interesting to explore the scope of further research on the possibilities of these antioxidants to ameliorate both COVID-19 and the disease-induced male reproductive dysfunctions.

## 2. COVID-19 and Male Infertility: Oxidative Stress (OS)–Inflammation Vicious Cycle

Oxidative stress occurs via a disrupted prooxidant–antioxidant balance that shifts towards prooxidant over-production [15]. Oxidative stress and inflammation are interlinked phenomena [14]. SARS-CoV-induced OS is mediated via various pathways. The virus evokes innate immune responses [14] triggering inflammation and excessive production of reactive oxygen species (ROS), eventually leading to OS [14]. A systemic ‘cytokine storm’ further elicits inflammatory responses leading to severe tissue injury [16].

The testicular microenvironment maintains immune homeostasis, where local innate immune responses are counteracted by an immune suppressive system [17]. Severe systemic inflammation via blood-borne dissemination of pathogens or via secondary inflammation, may adversely affect male reproductive functions [18,19].

**Figure 1:**
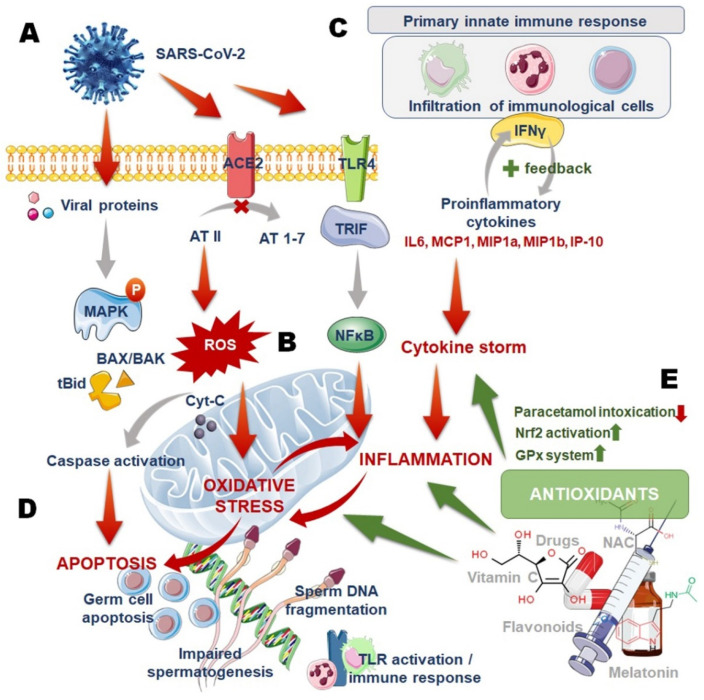
Mechanisms of SARS-CoV-2-mediated oxidative stress and male reproductive disruptions (**A**–**D**) and the roles of antioxidants in mitigating the damaging actions (**E**).

The high expressions of angiotensin-converting enzyme 2 (ACE-2) receptors in the testicular cells may indicate direct viral invasion in these cells [20]. Viral infections may act via specific host pattern recognition receptors (PRRs) [21]. Toll-like receptors (TLRs) are the best-characterized PRRs that can recognize viral proteins, nucleic acids, and oxidized macrophage phospholipids caused by high ROS levels [21,22]. Interestingly, Sertoli cells, Leydig cells, and spermatogonial cells express different TLRs [22]. Activations of TLRs may trigger myeloid differentiation primary response 88 (MYD88) and mitogen activated protein kinase (MAPK) pathways, activating the transcription factors, nuclear factor kappa-light-chain-enhancer of activated B cells (NFkβ), and interferon regulatory factor 3 (IRF3). This, in turn, upregulates transcription of inflammatory gene subsets including Sertoli cell immunoregulatory activin A [18]. The inflammatory mediators induce infiltration of activated leukocytes exaggerating generation of ROS and inducing testicular OS (Figure 1).

As SARS-CoV-2 shares a 79% nucleotide resemblance to SARS-CoV, it may utilize a similar primary immune-evasive strategy as reported for SARS-CoV [23]. SARS-CoV-induced OS leads to high release of macrophage-derived oxidized phospholipids [24], which can trigger cytokine overproduction and amplify the host inflammatory response via oxidant sensitive inflammatory pathways [24]. NF-kβ plays a central role in SARS-CoV infections [24]. It has binding sites in the promoter region of genes related to apoptotic elements and pro-inflammatory mediators. SARS-CoV 3CL^pro^ (a viral protease) causes a significant increase in ROS production, which in turn, activates NF-kβ-mediated cell apoptosis [21]. SARS-CoV acts through MAPK pathway that may act through Bax oligomerization to activate mitochondrial apoptotic pathways [24]. Peripheral blood mononuclear cells (PBMC) of SARS-CoV infected patients showed upregulation of inflammatory genes in response to OS [21], which also suggests that SARS-CoV infection triggers the vitiating loop of inflammation and OS. Moreover, this virus can potentially cause orchitis which may also induce OS. Finally, SARS-CoV-2 infection inflicts psychological stress that again paves the way for OS [25].

Secondary inflammation of SARS-CoV-2 may also affect testicular functions. Sertoli cells are sensitive to endogenous inflammatory mediators, most notably interleukin (IL)-1A, IL1B, tumor necrosis factor-α (TNF-α), nitric oxide, transforming growth factor B3 (TGF B3) and type 1 and type 2 interferons [18]. These molecules may extend their effects on Sertoli cells as well as mediate intercellular communication within the seminiferous epithelium [21]. Consequently, the presence of viral components or ligands of TLRs, increased levels of inflammatory cytokines within the testis, or reaching the testis via blood in case of progressive systemic inflammation, may severely impair testicular functions [26].

SARS-CoV-2-mediated disruption in male reproduction is also related to androgen synthesis. There are conflicting findings indicating that SARS-CoV-2 infection in males results in acute stage hypogonadism [27], which is associated with increased levels of pro-inflammatory cytokines, mainly IL-1β, IL-6, and TNF-α [26]. Inflammation-induced OS may also influence the endocrine regulation of male reproductive functions including steroidogenesis [28].

## 3. COVID-19, Oxidative Stress (OS) and Male Infertility: Role of Antioxidants

**Figure 2:**
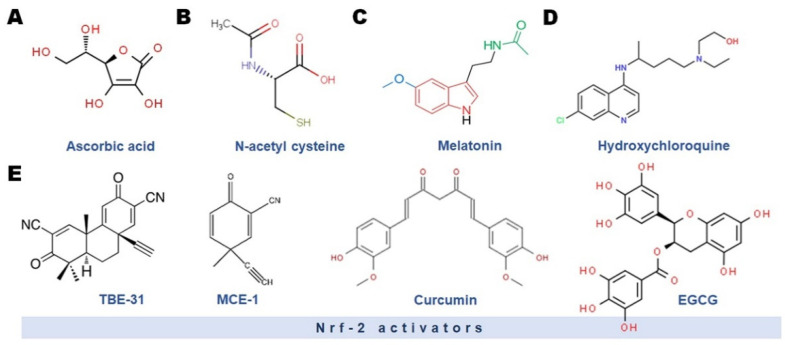
Most common antioxidants used in the management of SARS-CoV-2 infection.

In order to reduce the threat of SARS-CoV-2 infection and/or to be utilized as an adjuvant treatment in the case of severe COVID-19 forms, several therapeutic measures involving antioxidant(s) have been investigated or proposed (Figure 2).

### 3.1. Vitamin C

Given the intricate involvement of oxidant sensitive mechanisms in COVID-19-mediated male infertility, OS-targeted therapies may possibly lead to effective amelioration [29,30]. Early use of high-dose vitamin C may be beneficial in reversing these adverse effects. Vitamin C has been shown to benefit critical care management since it is a major component of the cellular antioxidant system [31,32]. A clinical study involving 146 patients with sepsis showed that intravenous high-dose vitamin C may be an effective treatment regime [33]. Vitamin C and sulforaphane have been shown to decrease OS-induced acute inflammatory lung injury [34].

Vitamin C is a key antioxidant in the testis and is particularly effective in neutralizing ROS and reducing sperm agglutination. It contributes electrons to redox systems, inhibits lipid peroxidation, recycles vitamin E, and protects DNA from peroxide radical damage [35] all of which can help preserve testicular cells from OS and also help reduce sperm DNA fragmentation (SDF). Additionally, it has been demonstrated that it increases serum testosterone levels in animals exposed to OS [36,37,38]. Thus, incorporating vitamin C in the therapy regimens of COVID-19 may benefit males with primary hypogonadism. In China, high-dose intravenous vitamin C was found to be beneficial in the treatment of 50 patients with moderate to severe COVID-19. An expert panel of National Institutes of Health has demonstrated that vitamin C dosage of 1.5 g/kg body weight is safe [39]. Given that high-dose vitamin C is regarded as safe, healthcare providers should benefit from these scientific and clinical observations. For COVID-19 male patients, well-designed clinical trials are required to investigate the efficacy of vitamin C therapy combined with standard treatment for resolving the systemic viral infection together with restoring male reproductive functions.

### 3.2. N-Acetyl Cysteine (NAC)

As a powerful antioxidant biomolecule, *N*-acetyl cysteine (NAC) may be used to combat the generation of ROS and, more significantly, the ‘cytokine storm’ that occurs in COVID-19 [40,41]. Similar to SARS-CoV, SARS-CoV-2 is thought to cause an immune response involving pro-inflammatory cytokines such as IL-1, IL-2, IL-4, TNFα, and IFNs. SARS-CoV infection inhibits type-I IFNs by inhibiting STAT1, antagonizing IFN. In SARS-CoV-2 infection, a cytokine storm causes delayed IFN response. *N*-acetyl cysteine may boost TLR-7 and mitochondrial antiviral signal protein signal cascades, restoring SARS-CoV-2-mediated type-I IFN production [40]. NF-κβ is a mediator of SARS-CoV-2 pathogenesis playing a central role in triggering a cytokine storm. However, in an in vitro influenza A and B model, NAC inhibited NF-κβ activation [42], replenishing thiol pools and the ROS scavenging mechanism. Decreased GSH levels and increased ROS production aid the progression of inflammatory diseases. A recent study reported an elevated ROS/GSH ratio in patients with severe COVID-19 infection compared to mild forms [43]. Since secondary immune responses elicited by systemic inflammation and OS may cause male reproductive dysfunction in COVID-19 patients [44], anti-inflammatory and antioxidant characteristics of NAC may protect the tissues from the oxidative damage.

Oral, intravenous, or inhaled NAC in patients with mild COVID-19 symptoms has been used as a less expensive clinical treatment. Clinical trials are carried out to find the safety and effectiveness of combination-treatment for ventilated COVID-19 patients by an FDA-approved drug, nebulized heparin-NAC by evaluation of pulmonary functions [45]. Another recent trial found that COVID-19 patients taking 6 g NAC intravenously daily had improved treatment outcomes [46,47]. Additionally, oral NAC (600 mg/day) could also prevent SARS-CoV-2 in people who are constantly exposed to the virus.

Thus, NAC is a potent COVID-19 therapeutic drug whose possible effectiveness in ameliorating COVID-19-mediated male reproductive impairment is an important research area to explore. It has been reported that NAC supplementation can improve sperm parameters and oxidative/antioxidant state in infertile men [48,49]. Due to its free sulfhydryl group, NAC reduces sulphide bonds in the cross-linked glycoprotein matrix in mucus. Owing to its ability to cleave viral disulfide bonds [50], NAC may also help inhibit SARS-CoV-2 invasion into testicular cells. In addition to being a potent anti-inflammatory and antioxidant, NAC maintains the thiol pool, which in turn regulates the redox state as it is an important substrate for glutathione synthesis [51,52]. The anti-inflammatory and antioxidant effects of NAC may contribute to preserving male reproductive functions from COVID-19 mediated damages.

### 3.3. Melatonin

Melatonin does not have direct anti-viral effects [53], but owing to its anti-inflammatory, antioxidant, and immune boosting properties [54,55], it can contribute to the treatment of SARS-CoV-2 infection. In addition, melatonin is a potent inhibitor of calmodulin, which is a critical intracellular component for the maintenance of ACE-2 on the cell membrane [56]. The multifunctional pineal hormone, melatonin, has been shown to reduce the symptoms of viral infections in some instances. After infecting mice with a virus (e.g., encephalitis), researchers found that using melatonin lowered the viral load in their blood and brain and reduced the severity of paralysis and death [57]. Recent studies using respiratory syncytial virus models found that melatonin was effective in decreasing acute lung oxidative injury, as well as pro-inflammatory cytokine production and inflammatory cell recruitment. These data, together with those previously presented by Reiter et al., provided evidence in favor of the use of melatonin in the treatment of viral infections including COVID-19 [53]. According to recent research, patients with obesity and diabetes, who are at high risk of severe inflammation and OS following infection with SARS-CoV-2, may benefit from the use of melatonin [58]. Moreover, it has been posited that children may not suffer from COVID-19 as much as their grandparents, as melatonin diminishes with age [59]. Melatonin influences male reproduction in three major ways. First, it modulates GnRH and LH secretion. Second, it controls testosterone biosynthesis and maturation of testes. Third, as an effective lipophilic and hydrophilic free radical scavenger, it protects against toxicants and inflammation [60,61,62]. Thus, melatonin is a potentially endogenous molecule to be studied extensively for its role in COVID-19 mediated male reproductive disruptions.

### 3.4. Selenium (Se)

Selenium (Se) deficiency may impact COVID-19 severity. A study conducted including 17 Chinese cities showed a positive correlation between COVID-19 prevalence and endogenous Se concentration, and Se insufficiency was linked to an elevated COVID-19 mortality risk [63]. Evidence suggests that the micronutrients zinc, Se, and vitamin D might be involved in the course and outcome of COVID-19 disease, but the number of studies undertaken in this area still remains inadequate. However, it was hypothesized that nutritional supplement(s) given during the early phases of infection may boost the host’s resistance to the infections [64]. The ebselen organoselenium compound reduces the activities of hydroperoxide- and peroxynitrite thereby mimicking the enzymes, glutathione peroxidase and peroxiredoxin. Ebselen forms a selenosulfide bond by reacting with several protein thiols, and this is attributed to its pleiotropic characteristics, mainly, antibacterial, antiviral, and anti-inflammatory. The main protease (M^pro^) of SARS-CoV-2 is a potential drug target, and among over 10,000 compounds that have been screened to identify the specific potent M^pro^ inhibitor, ebselen has emerged as one of the potent M^pro^ inhibitors [65,66]. In humans or other animals, the prime role of Se attributes to its antioxidant actions through Se-dependent enzyme glutathione peroxidase (GPx1). The GPx1 system protects the cells from OS mediated peroxidative damage to the membranes and organelles. Moreover, through this mechanism of action of Se, it has been found to improve semen parameters in infertile men and also increases pregnancy rates [67,68]. The connection between the GPx1 detoxifying system and the primary protease (M^pro^) of SARS-CoV-2 also represents a unique molecular target for COVID-19 [69]. Considering the beneficial impacts of Se on male fertility as well as on COVID-19 via similar mechanisms, it may be deemed that Se is a potential research candidate to be explored for male reproductive restoration in COVID-19-infected men.

### 3.5. Nrf-2 Activators and Flavonoids

S-protein of SARS-CoV-2 binds ACE2 receptors for its cellular entry followed by downregulation of endogenous anti-viral mechanism, activation of NF-κβ pathways, and production of pro-apoptotic proteins and ROS [70]. The nuclear factor erythroid 2-related factor-2 (Nrf2) is one of the main components mediating cellular oxidant resistance. To minimize the adverse effects of oxidant exposure, Nrf2 regulates the expression of numerous antioxidant response element (ARE)-dependent genes. Thus, Nrf2 activators have been conjectured to suppress the impacts of SARS-CoV-2 infection [70]. Nrf2-activators include curcumin, gingerol, capsaicin, epigallocatechin gallate (EGCG), genistein, lycopene, resveratrol, diallyl sulphide, phenethyl ester, indole-3-carbinol, and sulphoraphane. On the other hand, the synthetic Nrf2 activator PB125^®^ was found to downregulate 36 genes encoding cytokines such as IL-6, IL-1β, TNFα, and cell adhesion molecules as well as a group of IFN-induced genes [71]. Curcumin, a natural Nrf2 activator with minimal toxicity and antioxidant, and anti-inflammatory properties, has been proposed as a therapeutic agent for viral pneumonia and ALI/ARDS. By stimulating polymorphonulcear leukocytes’ (PMNs) apoptosis and neutralizing ROS, curcumin protects against inflammation and OS [72]. The antioxidant epigallocatechin-3-gallate (EGCG), found in green tea leaves, has been advocated as a supplement therapy for COVID-19 patients. Most of EGCG’s advantages come from its anti-fibrotic activity and ability to downregulate various inflammatory mediators [73]. The thiols glutathione (GSH) and NAC are also Nrf2 activators [74].

These compounds acting as Nrf-2 activators and flavonoids, owing to their anti-inflammatory, antioxidant, anti-apoptotic, and antiviral activities, are suitable candidates for nullifying impairing effects of oxidants on semen quality. The flavonoids, naringin, rutin, catechin, kaempferol, and quercetin were found to be ameliorative of sperm oxidative damage [75]. These may, therefore, be beneficial in the prevention or reduction of COVID-19 severity as well as its impacts on male reproduction.

### 3.6. Drugs

Antioxidant characteristics may be found in COVID-19 therapy drugs. Hydroxychloroquine is an old malaria drug now used to treat autoimmune diseases such as rheumatoid arthritis and systemic lupus erythematosus (SLE). Although hydroxychloroquine has several protective benefits, including antioxidant mechanisms [76], the same drug has been shown to exhibit oxidative characteristics due to a reduction in GSH levels [77]. Its antioxidant, immunomodulatory, and antiviral actions have been recommended in the 6th and 7th versions of Clinical Practice Guideline on COVID-19 in China [78]. Although paracetamol is used in COVID-19, it has been suggested to employ NAC as an adjuvant treatment, maybe in combination with other medications, at high intravenous dosages similar to those used as an antidote to paracetamol intoxication [77]. It is worth noting that paracetamol, which is the chosen medicine for symptomatic and domiciliary therapy of COVID-19 in its early stages, can deplete GSH, especially in individuals with a greater COVID-19 risk, raising the chance of severe COVID-19 forms [79]. In the case of extended administration of high dosages of this antipyretic and analgesic drug, it would be necessary to determine if NAC supplementation should be used regardless of COVID-19 [79]. It has been reported that excess dosages of paracetamol appear to alter semen quality, notably sperm morphology, and hence its capacity to fertilize. Paracetamol may have this impact on sperm quality by limiting testosterone synthesis, generating OS, inducing germ cell apoptosis, and lowering prostaglandins and nitric oxide production. It will be critical to carry out more studies, particularly clinical research, to corroborate these findings [80]. Moreover, given the ameliorating impacts of NAC on male fertility (as previously discussed), it may be assumed that in the treatment of COVID-19, if NAC is combined with the conventional paracetamol therapy, it may prevent adverse impacts of paracetamol upon semen quality and overall male reproductive health.

## 4. Conclusions

Oxidative stress is a prominent pathogenic mechanism in both chronic degenerative and infectious diseases. Oxidation, systemic inflammation, and immune response deficiency are all linked with COVID-19, that may adversely impact male fertility. Given the concerning global declining trend in male fertility, it is important to gauge the impact of the COVID-19 pandemic and its treatment regime on male reproductive health. The article has precisely discussed the use of several antioxidants in the treatment of COVID-19 and their possible roles in the amelioration of COVID-19-mediated disruptions in male fertility. There are few potential candidates, such as the vitamin C, NAC, flavonoids, and melatonin as well as Se, which should be essentially studied further for their abilities to address both COVID-19 and its impacts upon male reproductive functions.
